# Privacy Challenges to the Democratization of Brain Data

**DOI:** 10.1016/j.isci.2020.101134

**Published:** 2020-05-05

**Authors:** Nicole Minielly, Viorica Hrincu, Judy Illes

**Affiliations:** 1 Neuroethics Canada, Division of Neurology, Department of Medicine, University of British Columbia, 2211 Wesbrook Mall, Koerner S124, Vancouver, BC V6T 2B5, Canada

**Keywords:** Techniques in Neuroscience, Biotechnology, Bioethics, Bioengineering, Biodevices

## Abstract

Wearable devices that record brain signals may present privacy concerns for consumers. Industry leaders discussed four such concerns with us that pertain to data collection and management, user autonomy and information transparency, exceptionalism of brain data, and regulatory systems.

## Graphical Abstract

**Figure:**
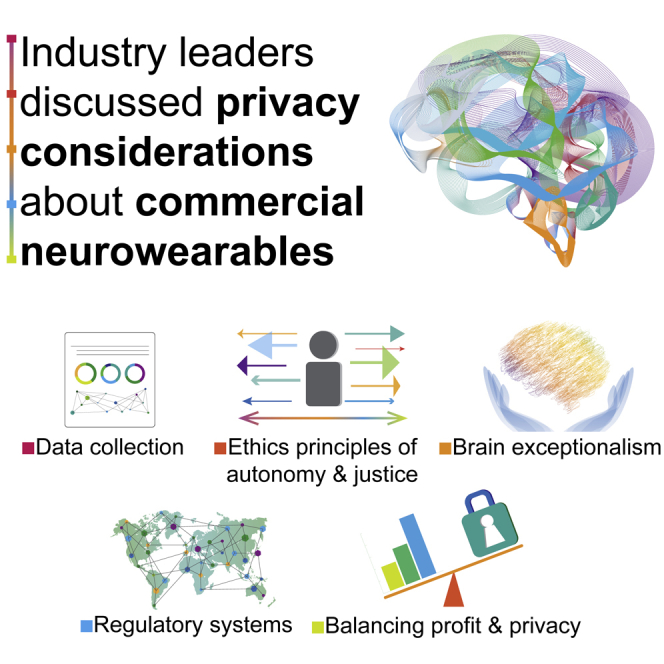

## Introduction

### What We’re Trying to Do Is to Democratize Access to Brain Health. (Interview 3)

Direct-to-consumer (DTC) wearable devices that record brain signals (neurowearables) are readily available on the consumer marketplace. According to market research by *Neurotech Reports*, the global neurotechnology product market is valued at approximately $9.1 billion and will grow to approximately $15.1 billion by 2024. These devices are marketed for wellness and enhancement among other human phenomena and aim to empower users with insights into their brain health (Coates McCall et al., 2019). In a sense, they are democratizing brain health.

High-profile cases such as the 2018 Facebook Cambridge Analytica scandal (Isaak and Hanna, 2018) and Project Nightingale (Schneble et al., 2020), in which user data were collected and analyzed without user consent, have made privacy of all forms of user data a visible issue. Compounding the challenge of privacy protections is the behavior of consumers who accept user agreements with little regard to their terms, thereby giving access to their brain data for mining, analytics, and purchase by third parties (Ienca et al., 2018, Kellmeyer, 2018). The collection of brain data potentially faces higher scrutiny because of its exceptional nature connected with personhood, agency, and decision-making (Ienca et al., 2018), and, as research has shown, the potential—whether reality or perception—to reveal personal insights (Martinovic et al., 2012, Wexler, 2019). Through a study we conducted with DTC neurowearable industry leaders, we observed that they too are thinking about key privacy issues and the lack of international standards. To our knowledge, no prior reports from this group have been published in either the peer-reviewed or gray literature.

## Methods and Results

Through a structured secondary analysis (Cabrera et al., 2015) of interviews with neurowearable company leaders or their senior level designates (Minielly et al., 2020), we identified privacy concerns that clustered into four major thematic categories: data collection and management, ethics principles, exceptionalism of brain data, and international policies, laws, and standards (Table 1).

**Table 1:** Four Thematic Categories about Neuroprivacy

Themes	
Data collection and management	*“The much bigger problem [than incidental findings] is the privacy problem […] how do you reassure your users that their data is protected, and […] that the rights are theirs and not someone else's to access the data.” (Interview 6)* *“We're likely to hit a place like most other industries where companies need to own the data in order to monetize and to do anything valuable with it. Not to say that they're directly selling the data, but there's a reason we collect it and it's to become a prominent player in using this data for intelligence purposes and not being able to do so would hinder our company's ability to exist […] That said, we shouldn't be allowed to do everything we want with the data.” (Interview 1)*
Ethics principles	“*You do this in a way that privacy is paramount and you're developing this with the end user's rights and autonomy as a guiding principle.” (Interview 6, on autonomy)* *“[…] most neurotech companies I talk to don't have privacy policies and the reason is it's not in our best interest to have privacy policies. […] we work better if we own everything and we kind of just need to tell the customers […] what they need to hear to be happy with that.” (Interview 1, on transparency)*
Exceptionalism of brain data	*“[…] people are a little more personal about brain data and as a result the privacy problem is more prominent with [them].” (Interview 1)* *“The question that always comes back to my mind is there something that is unique to this brain access that we have that tells us something that we can't otherwise – that someone couldn't otherwise determine on the basis of, like, digitally observing behavior. And I haven't seen anything of that sort in any consumer oriented neurotech.” (Interview 6)*
International standards, policies, and laws	*“The consumers have access to their own data. So, the policy of the company is we do not sell data to a third party. People have the option to share data with us anonymously, but should they share the data with us, this data is not sold to a third party and we are fully GDPR compliant globally.” (Interview 2)* *“The privacy legislation in different jurisdictions has never kept up with technology. So, we've very much had to take it upon ourselves to implement very strict privacy controls.” (Interview 12)*

Some quotes may have overlapping relevance to multiple themes. Ellipses are used to shorten quotes for clarity.

## Discussion

### Neuroprivacy Meets Neurowearables

Participants echoed many solutions previously recommended by researchers to address privacy challenges, such as the need to improve consent and autonomy of consumers and the importance of limiting data collection. They also described concerns about data management. In fact, protecting user data was discussed as a greater consideration than the topic of incidental findings and adverse events arising from device use, which was the original research question in the parent study. Participants suggested that discarding collected data may be one solution to mitigate risks to privacy, and although they also described that data ownership may be beneficial for company gains, they expressed that there should be boundaries to this right.

Consideration of the autonomy of users with respect to their ability and right to make decisions for themselves was raised as a particularly important feature of device development, especially in the context of discovery science and resulting commercialization. The benefits and pitfalls of transparency around data collection were a similarly substantial consideration. Participants expressed contrasting views regarding the exceptionalism of brain signals. Some, like Ienca and colleagues (Ienca et al., 2018, Ienca et al., 2019), noted the potentially exceptional and personal nature of neural signals; others, like Wexler, subscribed to the view that the exceptionalism of DTC neurowearable data may be more perception than reality (Wexler, 2019). Many participants noted the diversity of company policies and standards in use today, the time lag between technological innovation to regulation, and varying international regulations that can impede global industry best practices. They further emphasized differences in privacy standards between companies and privacy laws around the world and the importance of attention to precedents about privacy set by other wearable device industries.

### New Perspectives on Familiar Concerns

Although we have read about privacy concerns from researchers in the past, this is the first time that leaders within the neurowearable industry weigh into the discourse in an empirically guided way. Scholars have suggested that improved consumer data literacy, limited data online/cloud storage, federated learning to de-personalize data output, and data deletion after a pre-identified period of time may benefit the industry (Ienca et al., 2018, Kellmeyer, 2018, Kreitmair and Cho, 2017). Prior frustrations voiced by researchers were reiterated by participants. For example, although the development, testing and marketing of neurowearables may comply with international standards such as Canada's Personal Information Protection and Electronic Documents Act (PIDEDA) and Europe's General Data Protection Regulation (GDPR), their use transcends borders and differing regulations and laws globally create inefficiencies overall. The US medical sector-specific HIPAA does not apply to data from wearable brain devices today, despite murky lines between health and wellness (Kreitmair, 2019, Minielly et al., 2020).

Harmonized safeguards are needed to protect data collected from the brains of humans, and industry leaders recognize the importance of balancing these measures with the extraordinary speed of progress in brain research and development that is driving the lucrative innovations of the neurowearable industry. Given one description from a participant about how the industry operates, it seems that privacy concerns are not always addressed directly by DTC neurowearable companies, offering an explanation as to why we may be among the first to report them:

*Most companies I know don't have data privacy policies and don't put them in place purposefully to try and not bring that debate to light because there's no winning that debate. If the customers think of you as being a privacy-centric problem […] that sheds negative light on your marketing regardless of if you're the best, most perfectly handled privacy company ever. And so, it's kind of better that nobody ever talks about it. Potentially that's a problem. (Interview 1)*

Remaining silent about neuroprivacy is a risky way forward. Given many valuable insights we gained from industry leaders through this work, we would eliminate the hedge of *potentially* altogether from the equation.

### Conclusion

Leaders in the recording device industry are concerned about user data privacy. Tensions between balancing the collection of user data for commercial gains and the need to uphold safeguards for data privacy protection are a particular focus. Different regulatory systems compound the complexity of privacy considerations on the neurowearable landscape and could be alleviated, or at least mitigated, by coordination of international privacy standards.

### Limitations of the Study

We appreciate that views may vary based on an individual's position in a company, the size and location of the company, the stage of device development, and that privacy considerations for stimulating devices may be different than those for recording devices. We also acknowledge the limited N of the participant group.

### Resource Availability

#### Lead Contact

Judy Illes, CM, PhD jilles@mail.ubc.ca.

#### Materials Availability

This study did not generate new unique reagents.

#### Data and Code Availability

This study did not generate or analyze datasets or code.

## Methods

All methods can be found in the accompanying Transparent Methods supplemental file.

## Supplemental Information

Document S1. Transparent Methods

## References

[bib1] Cabrera L.Y., Beattie B.L., Dwosh E., Illes J. (2015). Converging approaches to understanding early onset familial Alzheimer disease: a First Nation study. SAGE Open Med..

[bib2] Coates McCall I., Lau C., Minielly N., Illes J. (2019). Owning ethical innovation: claims about commercial wearable brain technologies. Neuron.

[bib3] Ienca M., Haselager P., Emanuel E.J. (2018). Brain leaks and consumer neurotechnology. Nat. Biotechnol..

[bib4] Ienca M., Haselager P., Emanuel E.J. (2019). Reply to “separating neuroethics from neurohype”. Nat. Biotechnol..

[bib5] Isaak J., Hanna M.J. (2018). User data privacy: Facebook, Cambridge Analytica, and privacy protection. Computer.

[bib6] Kellmeyer P. (2018). Big brain data: on the responsible use of brain data from clinical and consumer-directed neurotechnological devices. Neuroethics.

[bib7] Kreitmair K.V. (2019). Dimensions of ethical direct-to-consumer neurotechnologies. AJOB Neurosci..

[bib8] Kreitmair K.V., Cho M.K., Illes J. (2017). The neuroethical future of wearable and mobile health technology. Neuroethics: Anticipating the Future.

[bib9] Martinovic, I., et al. (2012). On the feasibility of side-channel attacks with brain-computer interfaces. In: Proceedings of the 21st USENIX Security Symposium.

[bib10] Minielly N., Hrincu V., Illes J., Bard I., Hildt E. (2020). A view on incidental findings and adverse events associated with neurowearables in the consumer marketplace. DNB 3: Ethical Dimensions of Commercial and DIY Neurotechnologies.

[bib11] Schneble, C.O., Elger, B.S. and Shaw, D.M. (2020) ‘Google’ s Project Nightingale highlights the necessity of data science ethics review’, pp. 3–4. 10.15252/emmm.202012053. 10.15252/emmm.202012053 PMC7059004 32064790

[bib12] Wexler A. (2019). Separating neuroethics from neurohype. Nat. Biotechnol..

