# Supplementary material for: Privacy Challenges to the Democratization of Brain Data
Source: iScience. 2020 May 5;23(6):101134. doi: 10.1016/j.isci.2020.101134 (PMC7235278; doi:10.1016/j.isci.2020.101134)
Supplement: Document S1. Transparent Methods [file mmc1.pdf]

iScience, Volume *23*

Supplemental Information

Privacy Challenges to the Democratization of Brain Data

Nicole Minielly, Viorica Hrincu, and Judy Illes

SUPPLEMENTAL ITEMS

TRANSPARENT METHODS

Eight executives or their senior level representatives from DTC neurowearable companies that produce recording devices offered their time to speak with us. These semi-structured interviews took place in the context of larger study on incidental findings and adverse events involving representatives from both recording and stimulating device companies (Minielly et al., 2020). Interviews were conducted online via Zoom between August and November 2019 by either JI or NM and ranged between 11 and 35 minutes. We applied a structured secondary analysis of the data to identify privacy concerns (Cabrera et al., 2015).
